# Consent to data linkage in a large online epidemiological survey of 18–23 year old Australian women in 2012–13

**DOI:** 10.1186/s12874-019-0880-z

**Published:** 2019-12-11

**Authors:** Anna Graves, Deirdre McLaughlin, Janni Leung, Jennifer Powers

**Affiliations:** 1 Research Centre for Generational Health and Ageing, University of Newcastle, Newcastle, New South Wales Australia; 2 Bloomberg Data For Health Initiative, Global Burden of Disease Group, Melbourne School of Population and Global Health, University of Melbourne, Melbourne, Victoria Australia; 3 School of Psychology, The University of Queensland, Brisbane, Australia

**Keywords:** Cohort studies, Consent, Data collection, Data linkage, Health surveys, Opt-in consent, Online, Young women, Classification and regression trees

## Abstract

**Background:**

Consent to link survey data with health-related administrative datasets is increasingly being sought but little is known about the influence of recruiting via online technologies on participants’ consents. The goal of this paper is to examine what factors (sociodemographic, recruitment, incentives, data linkage information, health) are associated with opt-in consent to link online survey data to administrative datasets (referred to as consent to data linkage).

**Methods:**

The Australian Longitudinal Study on Women’s Health is a prospective study of factors affecting the health and well-being of women. We report on factors associated with opt-in consent to data linkage at the end of an online survey of a new cohort of 18–23 year old Australian women recruited in 2012–13. Classification and Regression Tree analysis with decision trees was used to predict consent.

**Results:**

In this study 69% consented to data linkage. The provision of residential address by the individual, or not (as a measure of attitudes towards privacy), was the most important factor in classifying the data into similar groups of consenters (76% consenters versus 47% respectively). Thereafter, for those who did not provide their residential address, the incentives and data linkage information that was offered was the next most important factor, with incentive 2: limited-edition designer leggings and additional information about confidentiality showing increases in consent rates over Incentive 1: AUD50 gift voucher: 60% versus 37%.

**Conclusions:**

In young Australian women, attitudes towards privacy was strongly associated with consenting to data linkage. Providing additional details about data confidentiality was successful in increasing consent and so was cohort appropriate incentives. Ensuring that prospective participants understand the consent and privacy protocols in place to protect their confidential information builds confidence in consenting to data linkage.

## Background

Large epidemiological surveys are increasingly seeking consent to link or match survey data with administrative datasets [1]. Linking to these datasets can substantially enhance the utility of the collected data and allow researchers to answer important questions which are not readily answerable through the use of survey data alone [2]. Although some large prospective studies rely on opt-out consent for these linkages, recent practice has seen increasing reliance on opt-in consent to linkage as this greatly reduces the onus on researchers to demonstrate the benefit to public interest in allowing opt-out consent.

A recent systematic review assessing consent proportions to data linkage found that the consent proportion varied from 39 to 97% [3]. However, none of the studies was conducted online. Still there was considerable heterogeneity among the studies reviewed and variations among the methods by which consent was obtained, i.e. ranging from a face-to-face interview to a mailed letter. Despite this, the method of obtaining consent did significantly affect the consent rate, with both the top (97%) and bottom (39%) scoring studies using face-to-face approaches to elicit consent [3]. A number of previous papers have reported differences between consenters and non-consenters across a range of variables including age, sex, race, area of residence, income, education and health status and, where an interview was conducted, attributes of the interviewer [4–8], although results have not been consistent. Other researchers have determined that likelihood to consent in face-to-face interviews was related to the salience of the linkage request, attitudes to privacy and community mindedness [2]. In a web survey of employment a small increase in consent rates was found when the time-saving benefit of linkage was mentioned [9].

It may be that these conflicting results reflect an underlying lack of understanding about the research process and the secondary use of health data. A number of studies have indicated that participants are more likely to consent if they have been provided with clear, easily understandable information about the importance of data linkage and they understand the issues involved [10, 11] . Exploration of any discrepancies between consenters and non-consenters is important to exclude the possibility that systematic differences may exist. The presence of these may compromise the researchers’ abilities to draw unbiased inferences from the linked datasets.

The use of the Internet and online technologies (such as Web-enabled phones) for conducting epidemiological surveys has recently been reported [1, 12, 13] and are both cost-effective and particularly suitable for younger participants [5]. However, there is relatively little information about how this modality affects participants’ consents to linking their survey data to administrative datasets. Online surveys in the Netherlands (such as the Longitudinal Internet Studies for the Social Sciences) have included a request for consent to data linkages [1], but these have been based on an opt-out model (implicit consent) rather than opt-in (explicit consent).

Both State and Federal agencies in Australia retain data for administrative purposes. In many instances, these are longitudinal and contain high quality information about large numbers of Australians. The Australian Productivity Commission provides reports to the Australian Government on measures to improve the productivity and economic performance of the country and has recommended that access to administrative data by academics and other researchers should be regarded as a Government priority [14]. Accordingly, when considering survey design for the recruitment of a new cohort of young women born in 1989–95, the Australian Longitudinal Study on Women’s Health (ALSWH) included a consent model which incorporated information about data linkage and a request that participants provide consent to directly link survey and individual level administrative records. The ALSWH has well-established privacy protocols covering the linking of participants’ data that are in accordance with Australian current best practice [15], and the ability to link survey data to administrative datasets has the potential to deliver substantial benefits while still protecting personal privacy.

Little research exists about differences between consenters and non-consenters to data linkage in online surveys and even less for opt-in consent. This paper tries to fill this gap by evaluating differences between young women who did and who did not provide consent to data linkage via an online survey.

## Methods

### Study design

The ALSWH is a prospective study of factors affecting the health and well-being of women. In 2010, the ALSWH was provided with funding by the Australian Government Department of Health to recruit a new cohort of 18–23 year old women. Women born between 1989 and 1995 (1989–95 cohort) were recruited via online surveys between 2012 and 2013. Open recruiting was conducted using a variety of methods: Facebook (including Facebook advertising), other Web activities (such as Twitter, Instagram, YouTube), referrals (emails, snowballing), traditional media (including flyers, posters, postcards), and a fashion promotion. The recruitment strategies are illustrated diagrammatically in the recruitment paper for this cohort [16].

Two incentives were offered for women to complete the online surveys. Incentive 1: women were offered the chance to win one of a hundred AUD50 gift vouchers. Incentive 2: an intensive advertising campaign offered a chance to win one of 2000 pairs of limited-edition designer leggings with a theme reflecting the respondents’ birth period. The leggings were very fashionable and highly desirable at the time of the survey.

Implicit consent to the use of survey data was assumed if a woman completed an online survey. However explicit consent was requested to link that data to administrative datasets. All participants were provided with information on the reasons for the data linkage consent request and why the Medicare Australia card number was required. When Incentive 2 was offered, if the respondent did not consent to data linkage additional information popped up giving her a chance to change her mind. This additional information included further reassurances that health records provided via data linkage are confidential, examples of the type of information that the data linkage would provide and a link to an infographic [17] illustrating how data is linked anonymously using keys.

The ethics committees of the University of Newcastle (H-2012-0256) and The University of Queensland (2012000950) approved the research protocol.

### Participants

Data for this paper were drawn from women born between 1989 and 1995 who responded to an online survey for the Australian Longitudinal Study on Women’s Health. Comparison with the 2011 Australian Census showed that women in the sample were broadly representative of women of the same age nationally (Census 49.0% versus ALSWH 52.6% aged 18–20; Census 74.5% versus ALSWH 75.0% living in major cities excluding missing data) although a higher proportion of women had post-school qualifications (Census 33.8% excluding missing data versus ALSWH 48.5%).

### Variables

#### Opt-in consent to data linkage

The outcome of consent examined in this study refers to the consent to data linkage, measured at the end of the online survey. Participants were asked for consent to data linkage with administrative datasets. They were not asked for consent to participate in the online survey, because implicit consent is assumed through the completion of the online survey. A total of 25,541 women completed the online survey, with the consent question at the end of the survey. Of these women, 17,684 (69%) consented and 7857 (31%) refused consent to data linkage.

#### Recruitment, incentive and information

The method of recruitment was assessed from the question ‘How did you hear about the Australian Longitudinal Study on Women’s Health survey?’ and the responses were classified: ‘Facebook’, ‘other web activities’, ‘referral’, ‘traditional media’ and ‘fashion promotion’. Incentives and information were: AUD50 gift vouchers and basic information about linkage or designer leggings and additional information about linkage.

#### Sociodemographic factors

The women were asked to provide information on their age, area of residence, highest educational qualification, ability to manage on income, relationship status and if they live with one or both parents, or with other adults. Age was categorized as ‘18 to 20’, ‘21 to 23’. Area of residence was categorized according to the Australian Statistical Geography Standard (ASGS) Remoteness Areas as ‘major cities’, ‘inner regional’, ‘outer regional’, and ‘remote or very remote’. A further category, ‘missing’ was added as 22% of values were missing for area of residence. Level of education was categorized into four groups: ‘less than Year 12’, ‘Year 12’, ‘certificate or diploma’ and ‘university’. Women’s ability to manage on their available income was based on responses provided on a five-point scale. Relationship status was categorized as partnered (married or cohabiting) or not partnered, including separated, divorced or widowed.

#### Health status

Assessment of general health was self-reported with the following question “How would you rate your health now?” This question is derived from the SF36 and has been shown to be a valid and reliable indicator of general health status [18]. The Kessler Psychological Distress Scale (K10) [19] is a short screening scale of non-specific psychological distress in the anxiety-depression spectrum. Consistent with previous usage, [20] K10 scores were categorised as ‘low distress’ (10 to 15), ‘moderate distress’ (16 to 21), ‘high distress’ (22 to 29) and ‘very high distress’ (30 to 50).

Women were also asked, “Have you ever been diagnosed with or treated for”: chronic conditions including diabetes, heart disease, hypertension, asthma, and cancer other than skin cancer. These were categorised as ‘no major condition’ or ‘any major condition’.

#### Health risk factors

Health risk factors included smoking (‘current smoker’ or not), alcohol consumption, body mass index and physical activity. Based on usual quantity and frequency of standard drinks consumed, weekly alcohol consumption was categorised as ‘never drink alcohol’, ‘1 to 7 drinks’, ‘8 to 14 drinks’ or ‘more than 14 drinks’ [21]. Body mass index was based on self-reported height and weight and categorised as ‘underweight’ (less than 18.5 kg/m^2^), ‘healthy weight’ (18.5–24.9 kg/m^2^), ‘overweight’ (25–29.9 kg/m^2^) or ‘obese’ (30 kg/m^2^ or more) [22]. Level of physical activity was classified as ‘inactive’, ‘low’, ‘moderate’ or ‘high’ based on how much time was spent walking briskly, and doing moderate and vigorous leisure activities in the last week [23].

### Statistical analysis

Percentage of consenters versus non-consenters was compared across recruitment method, incentive and information re consent, socio-demographic, and health status variables using chi-squared tests. The Breiman, Friedman, Olshen and Stone (BFOS) Classification and Regression Tree (CART) [24] method for building decision trees was used, following instructions to approximate this in SAS Enterprise Miner 14.1 [25]. The BFOS method recommends using validation data if the dataset is large enough, hence the data has been partitioned equally into training and validation data. CART starts with the root node containing all individuals in the dataset, with the tree built recursively, then trained and pruned automatically. All variables were included in the analysis. The Gini reduction method was used as the measure of node impurity to determine node splitting. The assessment method was selected to prune the fully-grown tree. This selects the smallest subtree with the best assessment measure value. The misclassification assessment measure, i.e. the lowest proportion of misclassified observations, is used for a categorical target variable.

## Results

In this study, 69% of 25,541 women consented to data linkage. Consent differed significantly by method of recruitment, with those women who were recruited via Facebook the least likely to provide consent (67%) while those women who were recruited via the fashion promotion most likely to consent (84%) (Table 1). Women who were offered leggings and additional information on data linkage were significantly more likely to consent to data linkage than those solely offered a cash incentive (79% versus 61%). Examination of the sociodemographic variables indicated that minor differences existed between consenters and non-consenters. Data were missing for less than 2% of sociodemographic variables with the exception of area of residence (missing for 22.7%). Women who did not provide area of residence were significantly less likely to provide consent (47% versus 76%).

Few differences were observed between health characteristics of consenters and non-consenters (Table 2).

**Table 1:** Demographic Characteristics of Consenters and Non-consenters (*N* = 25,541)

Variables	N		Consenters *N* = 17,684	Non-consenters *N* = 7857	Chi-square *p* value
Recruitment					
Facebook	20,120	%	67	33	< 0.01
Other Web activities	1032	%	70	30	
Referral	959	%	71	29	
Traditional media	562	%	77	23	
Fashion promotion	2842	%	84	16	
Incentive and data linkage information					
1. AUD50 with basic information	13,664	%	61	39	< 0.01
2. Leggings with basic and additional information	11,877	%	79	21	
Age group					
18 to 20 years	13,432	%	68	32	< 0.01
21 to 23 years	12,109	%	70	30	
Area of residence					
Major cities	14,800	%	76	24	< 0.01
Inner regional	3341	%	75	25	
Outer regional	1358	%	75	25	
Remote or very remote	237	%	70	30	
Missing area	5805	%	47	53	
Highest level of education					
Less than Year 12	2123	%	68	32	0.51
Year 12	11,014	%	69	31	
Certificate or diploma	6822	%	70	30	
University	5565	%	69	31	
Managing on available income is					
Impossible	1208	%	67	33	< 0.01
Difficult all the time	5253	%	71	29	
Difficult some of the time	9000	%	70	30	
Not too bad	7331	%	68	32	
Easy	2708	%	69	31	
Partnered					
No partner	18,577	%	69	31	0.02
Partner	6627	%	70	30	
Living with parents					
Yes	13,282	%	68	32	< 0.01
No	12,247	%	71	29	
Living with other adults					
Yes	4577	%	73	27	< 0.01
No	20,952	%	68	32	

Missing was less than 2% for consenters and non-consenters for all variables except area of residence.

**Table 2:** Health Characteristics of Consenters and Non-consenters (*N* = 25,541)

Variables	N		Consenters *N* = 17,684	Non-consenters *N* = 7857	Chi-square p value
Self-rated health					
Excellent	1554	%	67	33	0.02
Very good	8713	%	70	30	
Good	10,750	%	69	31	
Fair	3733	%	70	30	
Poor	788	%	66	34	
Psychological distress					
Low	5134	%	69	31	0.56
Moderate	7437	%	69	31	
High	7008	%	70	30	
Very high	5950	%	69	31	
Any major chronic conditions^a^					
Yes	7171	%	69	31	0.19
No	18,366	%	69	31	
Smoker					
Not a current smoker	20,410	%	69	31	0.98
Current smoker	5121	%	69	31	
Alcohol consumption					
Never drink alcohol	2139	%	65	35	< 0.01
1 to 7 drinks per week	20,419	%	70	30	
8 to 14 drinks per week	2004	%	70	30	
More than 14 drinks per week	969	%	68	32	
Body mass index (kg/m^2^)					
Underweight (< 18.5)	2051	%	69	31	0.88
Healthy weight (18.5–24.9)	14,711	%	69	31	
Overweight (25–29.9)	4988	%	69	31	
Obese (≥30)	3573	%	70	30	
Physical activity					
Inactive	1658	%	68	32	0.45
Low	6369	%	69	31	
Moderate	5283	%	70	30	
High	12,172	%	70	30	

Missing data were no more than 1% of all variables for consenters and non-consenters

^a^ defined as any of diabetes, heart disease, hypertension, asthma, cancer other than skin cancer

Table 3 shows the relative importance of potential splitter variables in the CART. Area of residence was the most important followed by incentive. Other variables held some significance in the unpruned tree construction, i.e. recruitment and managing on income, but were not in the final pruned CART.

**Table 3:** Variable Importance

Variables	Importance	Validation Importance	Ratio of Validation Importance to Training Importance
Area of residence	1.0000	1.0000	1.0000
Incentive and data linkage information	0.4435	0.5130	1.1566
Recruitment method	0.3564	0.4122	1.1566
Managing on available income	0.3324	0.3844	1.1566

Other potential explanatory variables with lower scores of importance were not included in this table

**Fig. 1:**
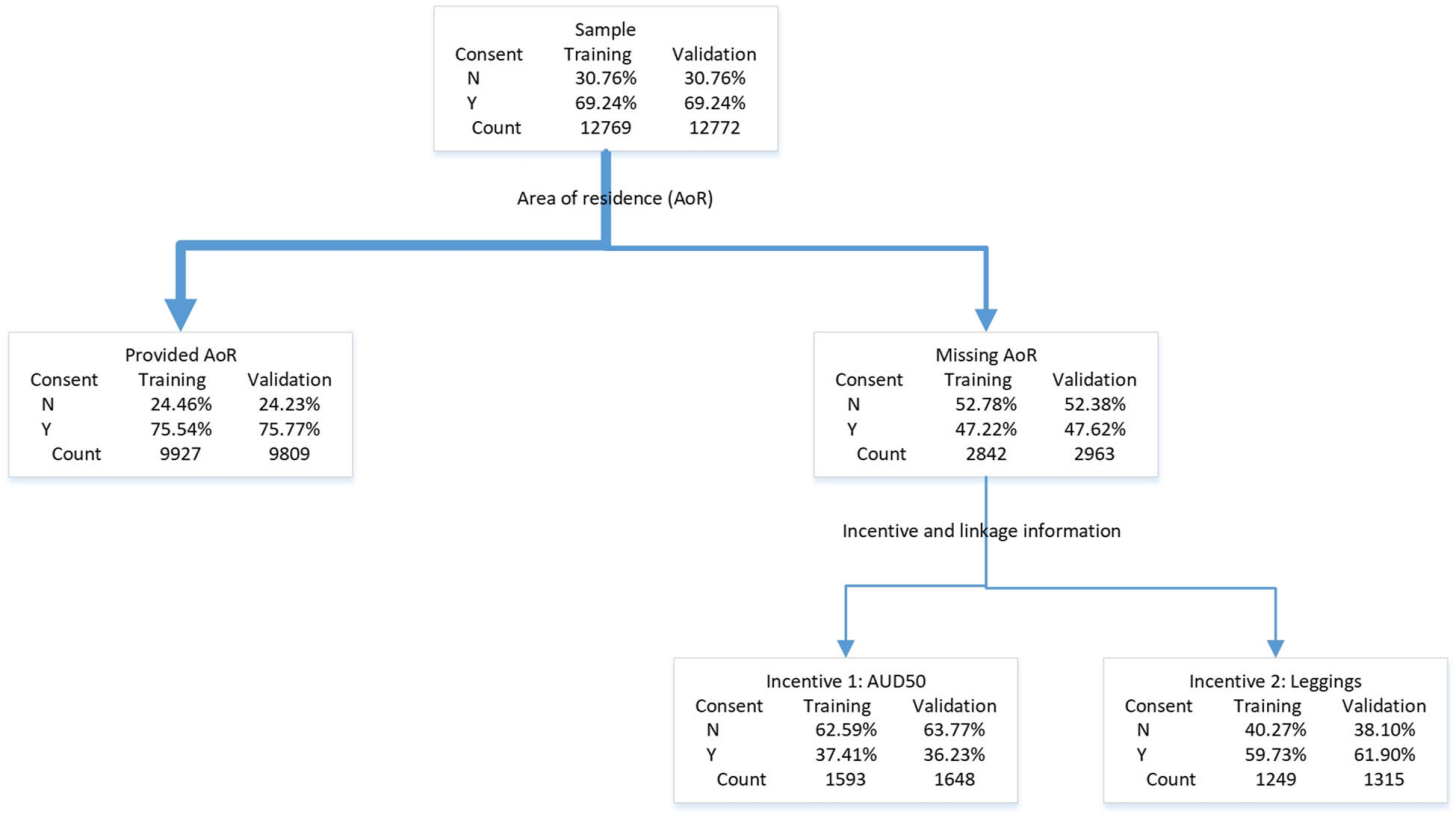
Classification tree for consent to data linkage

Figure 1 shows the pruned CART for consent to data linkage. Area of residence was the first splitter followed by incentive for women who did not provide area of residence, resulting in a tree with three terminal nodes. Other variables including recruitment method and managing on income did not lower the misclassification rate (0.27 for validation data) any further and were not included in the pruned tree.

## Discussion

More than two-thirds of the women who participated in this online survey provided opt-in consent to data linkage. Women who did not provide their area of residence were less likely to consent to data linkage. This may reflect a more cautious approach to divulging and sharing personal information among these young women. Previous research suggests that attitudes toward privacy and confidentiality are strongly related to non-consent [11]. For these women, it was apparent that consent differed by the incentive offered: women offered leggings and additional information about data linkage were more likely to consent than those offered a cash incentive and basic information about linkage.

In this study the consent question was at the end of the survey. The placement of consent has been identified as influencing response rates [26], although only one study was located which examined the consent placement in an online-administered survey [27]. In that study of German establishments, placement of the consent question at the beginning elicited higher consent rates than when it was placed at either the middle or the end of the survey. In those that utilised a telephone setting (e.g. computer-assisted telephone interviewing) the placement of the consent question at the beginning of the survey elicited higher consent rates [28]. However, the authors go on to aver that most linkage studies place the consent question at the end. A recent study on the placing of consent suggested that when this item is inserted at the beginning of a survey, it may impact on subsequent responses, although these measurement errors were confined to the recall of dates [29].

Some evidence exists that the wording of the linkage consent request influences participants’ choices. For example in a web survey of employment, income and expenditure, a small increase in consent rate was found when the time-saving benefit of linkage was mentioned [9]. In addition, a limited body of research suggests that assurances of confidentiality, identifying salient aspects of the linkage to respondents and providing some incentives may make respondents more likely to consent [11, 30, 31]. Participants in the current survey were provided with either a chance to win an AUD50 gift voucher and basic information about data linkage, or the opportunity to win a pair of leggings and additional information about data linkage if they refused consent. The results clearly indicate that the provision of additional information, together with the leggings, was associated with a higher rate of consent for those with privacy concerns. The leggings were highly desirable; however we were unable to ascertain if the provision of additional information alone or leggings alone would have resulted in a similar increase in consent. We were also unable to compare the use of incentives versus no incentives. This could be usefully explored in future data linkage studies.

Differences in the health characteristics of the consenting and non-consenting women were small, and consistent with earlier findings from a systematic review that reported some differences between consenters and non-consenters across all outcomes [6]. That same review also noted that there was a lack of consistency in the direction of differences across studies and in the magnitude of the association. The percentage of women who consented to this online survey was consistent with rates reported in a recent systematic review [3]. A potential limitation of the current study is the recruitment strategy, which was not based on a probability sample. However, comparison with the Australian Census suggests the women were broadly representative of Australian women of the same age, although they were more educated.

The relationship between higher education levels and active consent has been highlighted in a number of studies. For example, a randomised control trial in Australia [32], reported that both higher education and higher socio-economic status were associated with an affinity to consent. This was not consistent with the findings of this study however: while there were no differences on education level between consenters and non-consenters, small but significant differences were evident on the women’s ability to manage on available income.

One systematic review of participants’ attitudes to, and opinion of, linking research data to administrative data suggested that men and older respondents were more likely to provide consent [10]. However, this review also highlighted the general lack of knowledge about the process of data linkage and participants’ concerns about misuse and potential commercialisation of their data. This concurs with Australian research, which suggests that people are often not well-versed in the concepts of data linkage or de-identified data [32]. An exploration of reasons to consent or withhold consent found that most participants had a limited understanding of how data linkage worked and why they were being asked to provide consent [1]. In the same study comparison of online or mailed consent requests showed no differences in the percentage of consenters and non-consenters based on the mode of request [1]. A qualitative study of young adults [33] reported some confusion about various types of consent, with assumptions that opt-in consent equated to consent more generally. With opt-in methods, participants are generally provided with information and then asked if their data can be used for a specific purpose, as was the case for the current study. It may be that young people are more likely to consent with this method and this should be considered for future research.

## Conclusions

Increasingly, online surveys with data linked to administrative datasets, such as hospital and mortality records, are being utilised for large-scale epidemiological studies because of their cost-effectiveness and acceptability [34]. Despite this, scant research attention has been paid to the way in which consenters and non-consenters may differ and the implication this has for potential bias in survey results. This study contributes to the literature by identifying factors that may increase the rates of consenting to data linkage in young Australian women who participate in an online survey. Consent appears to be related to concerns about privacy and may be tempered by the provision of additional information about the linkage process and a desirable incentive. Ensuring that prospective participants understand what they are consenting to, if they elect to consent to data linkage, and the privacy protocols in place to protect their confidential information, may build confidence in the research process and enable researchers and policy makers to maximise the use of administrative datasets.

## Data Availability

The dataset supporting the conclusions of this article can be made available on request to the Australian Longitudinal Study on Women’s Health sph-wha@sph.uq.edu.au .

## Abbreviations

ALSWH: Australian Longitudinal Study on Women’s Health
AoR: Area of residence
ASGS: Australian Statistical Geography Standard
AUD50: 50 Australian dollars
BFOS: Breiman, Friedman, Olshen and Stone
CART: Classification and Regression Tree
K10: Kessler Psychological Distress Scale
SAS: Statistical Analysis System

## References

[1] Das M, Couper MP (2014). Optimizing opt-out consent for record linkage. JOS.

[2] Sakshaug JW, Couper MP, Ofstedal MB, Weir DR (2012). Linking survey and administrative records: mechanisms of consent. SMR.

[3] da Silva MEM, Coeli CM, Ventura M, Palacios M, Magnanini MMF, Camargo TMCR, Camargo KR (2012). Informed consent for record linkage: a systematic review. JME.

[4] Dunn KM, Jordan K, Lacey RJ, Shapley M, Jinks C (2004). Patterns of consent in epidemiologic research: evidence from over 25,000 responders. AJE.

[5] Harris ML, Loxton D, Wigginton B, Lucke JC (2015). Recruiting online: lessons from a longitudinal survey of contraception and pregnancy intentions of young Australian women. AJE.

[6] Kho ME, Duffett M, Willison DJ, Cook DJ, Brouwers MC (2009). Written informed consent and selection bias in observational studies using medical records: systematic review. BMJ.

[7] Knies G, Burton J, Sala E (2012). Consenting to health record linkage: evidence from a multi-purpose longitudinal survey of a general population. BMC Health Serv R.

[8] Patterson L, Cruise S, O’Reilly D (2013). Bias in consent to health data linkage: evidence from a UK cross-sectional survey [abstract]. J Epidemiol Community Health.

[9] Sakshaug J, Kreuter F (2014). The effect of benefit wording on consent to link survey and administrative records in a web survey. Public Opin Q.

[10] Hill EM, Turner EL, Martin RM, Donovan JL (2013). “Let’s get the best quality research we can”: public awareness and acceptance of consent to use existing data in health research: a systematic review and qualitative study. BMC Med Res Methodol.

[11] Sala E, Burton J, Knies G (2012). Correlates of obtaining informed consent to data linkage: respondent, interview, and interviewer characteristics. SMR.

[12] Couper M: Assessment of innovations in data collection technology for understanding society. In*.* Swindon: Economic and Social Research Council; 2012.

[13] Couper MP (2011). The future of modes of data collection. POQ.

[14] Productivity Commission (2010). **Annual report 2009–10**. In: *Annual Report Series*.

[15] Kelman CW, Bass AJ, Holman C (2002). Research use of linked health data—a best practice protocol. ANZJPH.

[16] Loxton D, Powers J, Anderson AE, Townsend N, Harris ML, Tuckerman R, Pease S, Mishra G, Byles J (2015). Online and offline recruitment of young women for a longitudinal health survey: findings from the Australian Longitudinal Study on Women’s Health 1989–1995 cohort. J Med Internet Res.

[17] Data Linkage Infographic [https://www.alswh.org.au/images/content/Resources/DataLinkageInfographic.jpg].

[18] Ware J, Kosinski M, Keller S (1994). SF-36 physical and mental health summary scales: a user's manual.

[19] Kessler RC, Andrews G, Colpe LJ, Hiripi E, Mroczek DK, Normand SLT, Walters EE, Zaslavsky AM (2002). Short screening scales to monitor population prevalences and trends in non-specific psychological distress. Psychol Med.

[20] Australian Bureau of Statistics: 4817.0.55.001 (2012). Information paper: use of the Kessler Psychological Distress Scale in ABS Health Surveys, Australia, 2007–08.

[21] Clemens SL, Matthews SL, Young AF, Powers JR (2007). Alcohol consumption of Australian women: results from the Australian longitudinal study on Women’s health. Drug Alcohol Rev.

[22] BMI classification [http://apps.who.int/bmi/index.jsp?introPage=intro_3.html].

[23] Brown Wendy J, McLaughlin Deirdre, Leung Janni, McCaul Kieran A, Flicker Leon, Almeida Osvaldo P, Hankey Graeme J, Lopez Derrick, Dobson Annette J (2012). Physical activity and all-cause mortality in older women and men. British Journal of Sports Medicine.

[24] Breiman L. FJH, Olshen R.a. and stone C.J.: classification and regression trees, 2nd edition edn. New York: Wadsworth International Group; 1984.

[25] Gordon L. Using Classification and Regression Trees (CART) in SAS Enterprise Miner™ For Applications in Public Health. SAS Global Forum 2013. 2013;(2013).

[26] Sala E, Knies G, Burton J (2014). Propensity to consent to data linkage: experimental evidence on the role of three survey design features in a UK longitudinal panel. Int J Soc Res Methodol.

[27] Sakshaug Joseph W, Vicari Basha J (2017). Obtaining Record Linkage Consent from Establishments: The Impact of Question Placement on Consent Rates and Bias. Journal of Survey Statistics and Methodology.

[28] Sakshaug J, Tutz V, Kreuter F (2013). Placement, wording, and interviewers: identifying correlates of consent to link survey and administrative data. SRM.

[29] Eckman Stephanie, Haas Georg-Christoph (2017). Does Granting Linkage Consent in the Beginning of the Questionnaire Affect Data Quality?. Journal of Survey Statistics and Methodology.

[30] Groves RM, Singer E, Corning A (2000). Leverage-saliency theory of survey participation: description and an illustration. POQ.

[31] Singer E, Von Thurn DR, Miller ER (1995). Confidentiality assurances and response: a quantitative review of the experimental literature. POQ.

[32] Berry Jesia G, Ryan Philip, Gold Michael S, Braunack-Mayer Annette J, Duszynski Katherine M (2012). A randomised controlled trial to compare opt-in and opt-out parental consent for childhood vaccine safety surveillance using data linkage. Journal of Medical Ethics.

[33] Audrey S, Brown L, Campbell R, Boyd A, Macleod J (2016). Young people’s views about consenting to data linkage: findings from the PEARL qualitative study. BMC Med Res Methodol.

[34] Rübsamen N, Akmatov MK, Castell S, Karch A, Mikolajczyk RT (2017). Comparison of response patterns in different survey designs: a longitudinal panel with mixed-mode and online-only design. ETE.

